# Reactive oxygen species in polycystic ovary syndrome: Mechanistic insights into pathogenesis and therapeutic opportunities

**DOI:** 10.1016/j.redox.2025.103776

**Published:** 2025-07-17

**Authors:** Huanju Liu, Lihao Jin, Xiaoya Wang, Junling Shi, Yujie He, Ningxia Sun, Fu Yang

**Affiliations:** a Department of Medical Genetics, Naval Medical University, Shanghai, China; b Institute of Genetics, International School of Medicine, Zhejiang University, Hangzhou, Zhejiang, China; c Women's Hospital, Zhejiang University School of Medicine, Hangzhou, Zhejiang, China; d Center for Reproductive Medicine, Second Affiliated Hospital of Naval Medical University, Shanghai, China

**Keywords:** Reactive oxygen species, Polycystic ovary syndrome, Metabolism, Targets

## Abstract

Polycystic ovary syndrome (PCOS), a common yet intricate endocrine disorder, presents persistent clinical challenges due to its complex pathogenesis. Reactive oxygen species (ROS), highly reactive molecules, have emerged as critical contributors to PCOS development and represent potential therapeutic targets. This review comprehensively explores the diverse roles of ROS in PCOS pathogenesis, particularly their contributions to metabolic dysfunction and hormonal imbalance. It further assesses the therapeutic potential of ROS-targeted interventions, summarizing antioxidant therapy advancements, underlying mechanisms, and current challenges. By consolidating recent research, this review highlights the profound significance of ROS in PCOS, aiming to inspire innovative pathogenesis research, improve clinical management, and establish a robust theoretical foundation for more effective PCOS treatment strategies.

## 1 Introduction

Polycystic ovary syndrome (PCOS) stands as one of the most prevalent endocrine disorders affecting women of reproductive age, affecting between 5 % and 20 % of this population depending on diagnostic criteria [1]. The syndrome is clinically characterized by hyperandrogenism, ovulatory dysfunction, and polycystic ovarian morphology, making it a primary contributor to anovulatory infertility, accounting for approximately 80 % of such cases. Beyond its reproductive impact, PCOS is strongly associated with a spectrum of metabolic disturbances, including insulin resistance, dyslipidemia, and obesity [2].

Emerging evidence points to mitochondrial dysfunction and oxidative stress as pivotal factors in the initiation, progression, and overall pathophysiology of PCOS [3,4]. Reactive oxygen species (ROS)—including the superoxide anion (O_2_^−^), hydrogen peroxide (H_2_O_2_), and hydroxyl radical (·OH)—are natural byproducts of aerobic metabolism. At physiological concentrations, these molecules are essential signaling components involved in critical cellular processes like cell signaling, immune responses, and cell cycle regulation [5]. However, when the balance between ROS production and antioxidant defenses (e.g., superoxide dismutase (SOD) and glutathione peroxidase (GPx)) is disrupted, oxidative stress occurs.

In individuals with PCOS, elevated levels of ROS, alongside increased markers of lipid peroxidation such as malondialdehyde (MDA), have been consistently documented in both serum and follicular fluid (FF), underscoring the direct relevance of oxidative stress in this condition [6,7]. Excessive ROS can profoundly impair ovarian function by interfering with key hormonal pathways crucial for follicular development and ovulation. Specifically, ROS disrupt the actions of follicle-stimulating hormone (FSH) and luteinizing hormone (LH), and can induce apoptosis in ovarian granulosa cells (GCs), thereby impeding proper follicular maturation [5,8]. Moreover, oxidative stress exacerbates insulin resistance through the activation of serine kinases that impair insulin signaling and damage β-cells, while also modulating enzymes such as cytochrome P450 17α-hydroxylase (CYP17A1) to promote hyperandrogenism [9,10]. Obesity, a frequent comorbidity in PCOS, further amplifies ROS production via dysfunctional adipose tissue metabolism, creating a feedback loop that intensifies the disorder's severity [11,12].

The intricate interplay between ROS and the multifaceted clinical manifestations of PCOS necessitates a comprehensive understanding of their molecular connections. The various clinical presentations of PCOS—reproductive, metabolic, and hormonal—are not isolated phenomena but are deeply interconnected through the central role of ROS. Elevated ROS levels arising from metabolic disruption can directly impair ovarian function; in turn, ovarian dysfunction exacerbates systemic oxidative stress, creating a self-reinforcing, pathogenic feedback loop. Drawing on studies published from 2020 to March 2025, this review aims to provide a detailed analysis of ROS-driven mechanisms and identifies potential therapeutic targets in PCOS. Our analysis is supported by newly compiled tables summarizing recent findings on ROS biomarkers, underlying mechanisms, model induction, and therapeutic interventions in cellular, animal, and human studies. By consolidating this research, we provide a nuanced perspective on how oxidative stress functions as both a biomarker and a mediator of PCOS, ultimately informing the development of more targeted and effective therapeutic strategies.

## 2 Theoretical foundations of ROS and PCOS

### 2.1 Etiological and clinical risk factors of PCOS

PCOS is a complex endocrine and metabolic disorder that significantly impacts women's health, with abnormalities including insulin resistance, hyperandrogenism, and irregular menstrual cycles. These issues can lead to severe long-term complications, including type 2 diabetes, cardiovascular disease, infertility, and psychological issues like anxiety and depression. For instance, PCOS significantly increases cardiovascular risk, predisposing individuals to conditions such as hypertension (with a risk ratio of 1.75), type 2 diabetes (risk ratio of 3.00), higher total cholesterol, lower high-density lipoprotein cholesterol (HDL-C), and an increased risk of non-fatal cerebrovascular events [[13], [14], [15]]. This highlights that PCOS is not solely a reproductive disorder but a systemic metabolic condition with significant public health implications, underscoring the urgency for early diagnosis and effective management strategies that target underlying drivers like oxidative stress.

PCOS exhibits marked heterogeneity in diagnostic criteria, which profoundly influences prevalence estimates and clinical management. The 1990 NIH criteria required both oligo- or anovulation and clinical/biochemical hyperandrogenism—excluding polycystic ovarian morphology (PCOM)—yielding prevalence estimates around 5–8 %. The 2003 Rotterdam criteria broadened the definition: two of three features—oligo/anovulation, hyperandrogenism, or PCOM—are sufficient for diagnosis. This expansion added two non-NIH phenotypes (hyperandrogenism + PCOM and ovulatory dysfunction + PCOM), inflating prevalence estimates to approximately 7–15 %, and even up to 21 % depending on population [2,16].

The inconsistency across diagnostic frameworks underscores the challenges in generalizing research findings, prescribing treatments, and interpreting epidemiological data. For instance, studies focused on androgen-excess phenotypes may not translate to normoandrogenic individuals, and comparing trials becomes complicated by varied phenotype inclusion. Although the AE-PCOS Society's criteria mandate hyperandrogenism for diagnosis, they've seen limited adoption [17]. Accurately specifying phenotypes in research and clinical settings is essential to clarify risk stratification, optimize interventions, and enhance comparability across studies.

Endocrine risk factors for PCOS include exposure to high levels of androgens and anti-Müllerian hormone (AMH) in utero, environmental pollutants, gut dysbiosis, neuroendocrine alterations (particularly hypothalamic-pituitary-ovarian (HPO) axis dysfunction manifesting as an elevated LH/FSH ratio), and lifestyle choices [18]. Metabolic risk factors for PCOS, such as insulin resistance and obesity, play a significant role in the disorder's development and management. Insulin resistance can lead to hyperinsulinemia, affecting the HPO axis, increases ovarian androgen production, and decreases follicular maturation and sex hormone-binding globulin (SHBG) binding [2]. Both endocrine and metabolic risk factors in PCOS have been mechanistically linked to ROS overproduction [5]. Therefore, investigating subcellular dysfunction, particularly mitochondrial abnormalities, is critical for understanding how these risk factors converge to drive oxidative stress and metabolic derangements.

### 2.2 Pathophysiological roles of ROS

#### 2.2.1 Different sources of ROS in PCOS

**Fig. 1:**
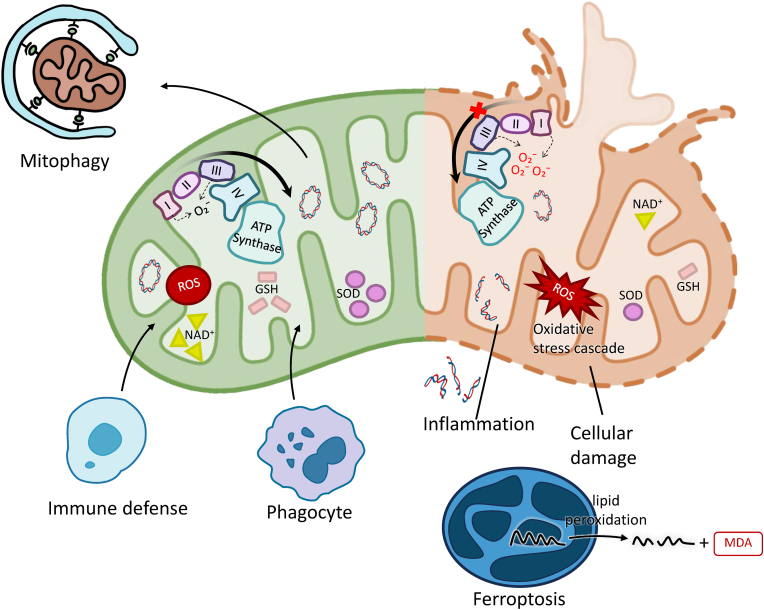
Physiological and Pathological Roles of ROS. The left panel depicts physiological ROS: balanced by antioxidants (SOD, GSH), ROS support essential processes like immune defense (phagocyte) and mitophagy. The right panel illustrates ROS dysregulation: mitochondrial dysfunction (e.g., Complex III block) leads to excessive ROS, triggering oxidative stress, cellular damage, inflammation, and ferroptosis (via lipid peroxidation and MDA).

ROS are highly reactive molecules generated mainly by the mitochondrial respiratory chain and NADPH oxidase during aerobic metabolism (Fig. 1). Approximately 2 % of oxygen is converted into ROS, notably the O_2_^−^, which serves as a precursor to other ROS. Specifically, electron leakage occurs at Complex I (flavoprotein domain) and Complex III (semiquinone radical binding site) of the mitochondrial electron transport chain, leading to O_2_^−^ production. In PCOS, dysfunctional mitochondria generate higher amounts of ROS within this electron transport chain, contributing significantly to the overall oxidative burden [19].

Beyond mitochondria, other enzymatic sources of ROS contribute to cellular oxidative stress. NADPH oxidases are significant membrane-bound enzymes that produce superoxide in the extracellular space by transferring electrons from the cytosol. In PCOS patients, the induced activity of membrane NADPH oxidase amplifies ROS production and sustains the inflammatory state observed in the condition. Hyperglycemia, a consequence of insulin resistance, and increased fatty acids, often seen in obesity, directly activate NADPH oxidase, thereby linking this enzyme to key aspects of PCOS pathogenesis [20].

Xanthine oxidase is another enzymatic source of ROS, contributing to oxidative stress alongside other redox reactions that occur in the endoplasmic reticulum. Furthermore, cytochrome P450 (CYP450) enzymes, a superfamily of monooxygenases, are involved in metabolizing various endogenous compounds, including lipids, proteins, and hormones [21]. While their primary role is metabolism, CYP450 enzymes can generate ROS as byproducts during their catalytic cycle. Dysregulation of these enzymes can lead to endocrine disorders, and in the context of PCOS, the enzyme CYP17A1 is crucial for androgen synthesis. Its enhanced activity by ROS contributes to hyperandrogenism, potentially forming a feedback loop where ROS generated by CYP450 activity further promotes androgen production.

The detailed elucidation of these multiple ROS sources—mitochondria, NADPH oxidase, xanthine oxidase, and CYP450 enzymes—reveals that oxidative stress in PCOS is a complex, multi-factorial burden, not attributable to a single pathway. This understanding suggests that effective therapeutic strategies must consider targeting multiple points of ROS generation or enhancing systemic antioxidant capacity to achieve comprehensive redox balance. Identifying the specific, dysregulated ROS-generating pathways in individual PCOS patients could pave the way for precision medicine, where therapies are tailored to inhibit the most active or relevant ROS sources in a particular individual. This approach could lead to more effective and less toxic interventions compared to broad-spectrum antioxidant supplementation, moving towards a diagnostic-driven, personalized therapeutic strategy.

#### 2.2.2 Redox homeostasis of ROS

At controlled levels, ROS function as essential signaling molecules. For instance, H_2_O_2_ can modify cysteine residues on proteins, thereby regulating the activity of kinases, phosphatases, and members of the mitogen-activated protein kinase (MAPK) family, which are pivotal for cell proliferation, differentiation, and apoptosis. Additionally, ROS are indispensable for immune defense; during the respiratory burst, phagocytes generate ROS to kill pathogens and stimulate cytokine release, thereby enhancing the inflammatory response and pathogen clearance [22,23]. Across species, ovulation requires ROS-mediated matrix metalloproteinase activation to drive follicular rupture and cumulus expansion, with evolutionary divergence in upstream triggers (*Drosophila*: NOX/octopamine; mammals: LH) [24,25]. These roles of ROS highlight their importance not only in normal cellular functions but also in the body's defense mechanisms, emphasizing the need for a delicate balance to prevent oxidative stress and associated diseases.

When cellular ROS production surpasses the capacity of antioxidant defenses (Fig. 1), oxidative stress damages lipids, proteins, and DNA—evident in lipid peroxidation marker MDA, compromised cell membranes, protein oxidation, and mutagenic changes [26,27]. In PCOS, this oxidative damage—exacerbated by impaired mitochondrial function—reduces the activities of key antioxidant enzymes such as manganese superoxide dismutase, SOD2 and GPx, leading to ROS accumulation and a vicious cycle of mitochondrial dysfunction, energy failure, disrupted oocyte maturation, and impaired steroidogenesis [7,[28], [29], [30]]. This cascade contributes to ovarian dysfunction in PCOS by interfering with follicular development, insulin signaling, and inflammation [21,31,32], highlighting the critical need to maintain redox balance for reproductive and metabolic health.

To counteract ROS generation, cells possess a robust antioxidant defense system (Fig. 1). This system comprises enzymatic antioxidants, such as SOD, catalase, and GPx, as well as non-enzymatic antioxidants like glutathione (GSH), vitamins C and E, and carotenoids. GCs adaptively increase the expression of Nrf2, a master transcriptional factor regulating antioxidant proteins, to maintain efficient ROS removal [33]. Additionally, early oocytes enhance their longevity by eliminating mitochondrial complex I—a major ROS generator—thereby reducing oxidative damage and activating the mitochondrial unfolded protein response [34]. These mechanisms of ROS homeostasis are crucial for preventing oxidative stress and maintaining cellular homeostasis, which in turn governs fertility [35,36]. However, in PCOS, these crucial antioxidant defenses are often compromised, leading to an imbalance that favors oxidative stress.

## 3 Mechanisms of ROS in PCOS pathogenesis

### 3.1 Oxidative stress in PCOS: clinical associations and biomarkers

PCOS patients manifest as elevated serum MDA levels—approximately 30 % higher than in healthy controls [37,38]—and reduced activities of key antioxidant enzymes such as SOD and GPx, alongside diminished nitric oxide (NO) levels [21,39], which collectively impair cellular capacity to neutralize ROS and disrupt vascular and endocrine functions. This oxidative stress exacerbates mitochondrial dysfunction in ovarian GCs, characterized by structural abnormalities like swelling and disrupted cristae, decreased mitochondrial membrane potential, and compromised ATP production, further impairing oocyte maturation and steroid hormone synthesis. Additionally, defects in mitochondrial DNA (mtDNA) replication or the accumulation of mutations contribute to the pathogenesis of PCOS by reducing activities of respiratory chain complexes I, III, and IV, lowering expression of key glycolytic enzymes [40], and causing NAD^+^ deficiency, all of which compromise cellular energy metabolism and predispose cells to apoptosis via the opening of permeability transition pores [[41], [42], [43], [44]]. These multifaceted disruptions underscore the critical role of oxidative stress and mitochondrial dysfunction in the pathophysiology of PCOS.

Disrupted mitochondrial dynamics—such as dihydrotestosterone (DHT)-induced ROS aggregation that perturbs mitochondrial fission [45], further promote GC apoptosis. Altered signaling pathways, including reduced Phosphoinositide 3-Kinase (PI3K) activity [46] and upregulated SREBF2/ALOX15 (Arachidonate 15-Lipoxygenase) expression [47], exacerbate lipid peroxidation and deplete intracellular antioxidants. Emerging biomarkers like elevated CD44 (Cluster of Differentiation 44) [48], dysregulated miR-196b-5p [49], and changes in the YTHDF2/MSS51 axis [50] further highlight the complex metabolic derangements and ferroptosis resistance present in ovarian tissue. Moreover, increased pro-inflammatory cytokines (IL-6, IL-1β, TNF-α) [51,52], elevated ROS levels in ovarian tissues [53,54], MDA-linked neural alterations [55], and altered Asymmetric Dimethylarginine-Dimethylarginine Dimethylaminohydrolase 1 (ADMA-DDAH1) axis signaling [56] collectively underscore the multifaceted role of oxidative stress in PCOS pathogenesis.

Various biomarkers summarized in Table 1 indicate increased oxidative stress in PCOS. These include elevated follicular fluid arachidonic acid (C20:4n6) [57], increased follicular fluid bilirubin [58], and high levels of follicular fluid MDA/8-OHdG [59]. These biomarkers are not merely indicators of oxidative stress; they correlate with significant clinical outcomes such as poor oocyte quality, impaired energy metabolism in GCs, and poor in vitro fertilization outcomes. The presence of these biomarkers not only illuminates the underlying mechanisms of ROS-mediated ovarian dysfunction but also offers potential targets for therapeutic intervention and provides a more dynamic and personalized approach to PCOS management, enabling earlier diagnosis, more precise risk stratification, and real-time assessment of treatment efficacy.

**Table 1:** Biomarkers and clinical associations.

Biomarker	Association with PCOS	Clinical Relevance	Ref
↑ CD44	Linked to ferroptosis resistance in ovarian tissue	Potential therapeutic target for ferroptosis	[48]
↓ DDAH1 activity	DDAH1 increases SOD1 expression and SOD activity	Alternative pathways for ovarian apoptosis in PCOS	[56]
↑ FF AA (C20:4n6)	Induces oxidative stress and ↑GDF15 in GCs	Correlates with poor oocyte quality	[57]
↑ FF Bilirubin	Correlates with ↓antioxidants and ↑ NO metabolites	Predicts poor in vitro fertilization outcomes	[58]
↑ FF MDA/8-OHdG	Marker of lipid peroxidation and DNA damage in obese PCOS patients	Linked to impaired energy metabolism in GCs	[59]
↑ IL-6	↑IL-6 in DHEA-induced PCOS rats with anxiety/depression	Links inflammation to neurobehavioral symptoms	[55]
↑ MDA	Correlates with structural brain changes and emotional dysfunction in PCOS rats	Marker of lipid peroxidation and neural damage	[55]
↓ miR-196b-5p	Regulates oxidative stress and glucose uptake in GCs	Diagnostic marker for GC dysfunction	[49]
↓ Mitochondrial Membrane Potential (ΔΨm)	Reduced ΔΨm in oocytes correlates with Foxo1-mediated DNA damage	Predicts oocyte apoptosis in PCOS	[7]
↓ NAD^+^	Reduced NAD^+^ in GCs correlates with ROS accumulation and fibrosis	Potential therapeutic target for ovarian dysfunction	[28,42]
↓ PI3K Activity	PI3K inactivation exacerbates ROS-induced GC apoptosis	Marker of metabolic dysfunction in PCOS	[46]
↑ Pro-inflammatory Cytokines	IL-6, IL-1β, TNF-α elevated in serum/FF of PCOS patients	Links oxidative stress to chronic inflammation	[51,52]
↑ ROS	Linked to androgen hypersecretion and fibrosis	Predicts oxidative stress severity	[53,54]
↑ ROS aggregation	DHT-induced ROS aggregation disrupts mitochondrial dynamics	Associated with GC apoptosis	[45]
↑ SREBF2/ALOX15	Increases lipid ROS and depletes GSH	Indicates lipid peroxidation in GCs	[47]
↑ TFRC (Transferrin Receptor)	Mediates iron-dependent ROS → ferroptosis in GCs	Potential target for mitigating ovarian cell death	[60]
↑ YKL-40 (Chitinase-3-like protein 1)	Correlates with oxidative damage in GCs	Therapeutic target for GC protection	[61]
↑YTHDF2, ↓MSS51	Links to mitochondrial dysfunction in GCs	Predictors of metabolic dysregulation in PCOS	[50]

### 3.2 Impact of ROS on follicular development

ROS play a dual role in follicular development. Under physiological conditions, moderate ROS levels serve as critical second messengers—activating pathways such as MAPK to promote GC proliferation and support normal ovulation [62,63]. However, excessive ROS levels result in ovarian cells bearing the brunt of oxidative damage. Hyperandrogenic conditions induced by agents such as DHEA or DHT, along with dietary factors like high‐fat diets, elevate ROS levels. This pathological overabundance triggers lipid peroxidation, depletes key antioxidant enzymes, and initiates ferroptosis and apoptosis, thereby impeding the follicular rupture necessary for oocyte release and driving anovulatory infertility [52,57,64].

At the subcellular level, ROS compromise mitochondrial integrity by dissipating membrane potential and opening the mitochondrial permeability transition pore. The ensuing cytochrome C efflux activates caspase-9 and caspase-3, while ROS-induced DNA damage stabilizes p53, shifts the Bax/Bcl-2 balance toward apoptosis [65], and upregulates Fas/FasL signaling [66]. Concurrent activation of matrix metalloproteinases further degrades extracellular matrix components, undermining follicular architecture and oocyte quality [7,67]. Collectively, these events collapse GC viability, curtail energy production via diminished glycolysis and ATP synthesis, and halt proper follicular maturation.

**Fig. 2:**
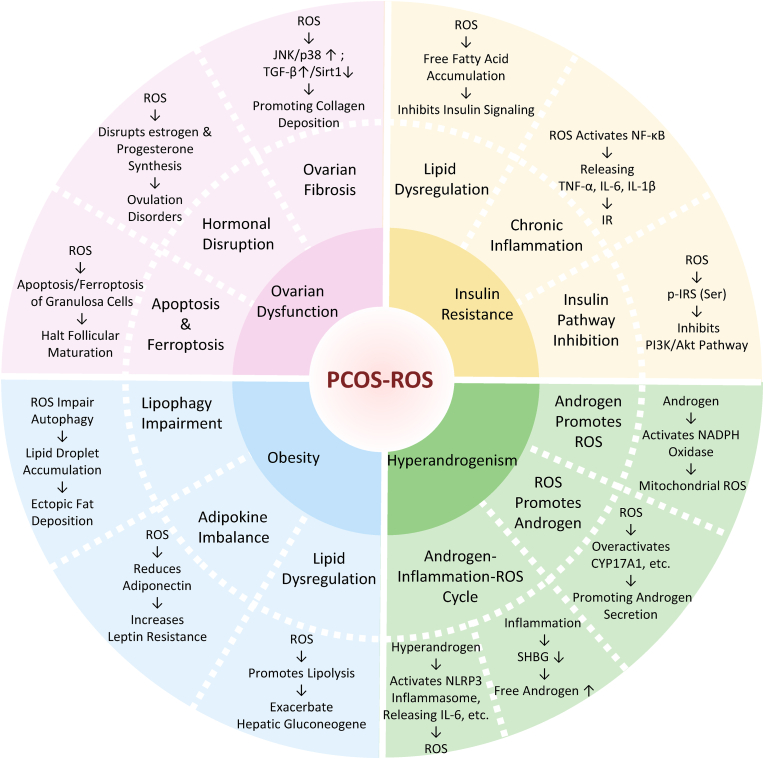
Mechanisms of ROS in PCOS Pathogenesis. This schematic illustrates the central role of ROS in driving key pathological features of PCOS. The circular framework highlights ROS as a hub interacting with four major PCOS phenotypes.

Beyond direct cellular effects, oxidative stress in PCOS perturbs systemic endocrine and metabolic networks. Elevated ROS disrupt HPO axis feedback—altering Gonadotropin-releasing hormone (GnRH) pulsatility and skewing the LH/FSH ratio—thereby exacerbating follicular arrest. The cumulative loss of GC function culminates in impaired estrogen and progesterone synthesis, reinforcing the hormonal imbalances characteristic of PCOS [63,68]. Encouragingly, targeted antioxidants and signaling modulators—such as lysophosphatidic acid, RAGE-inhibitory peptides, auraptene, and melatonin—have demonstrated efficacy in restoring mitochondrial health, bolstering endogenous antioxidant defenses, and enhancing fertilization outcomes [[69], [70], [71], [72]]. These interventions underscore the therapeutic promise of mitigating oxidative stress to rescue follicular development and improve fertility in women with PCOS (see Fig. 2 for a schematic overview).

### 3.3 Interaction between ROS and hyperandrogenism

Hyperandrogenism is a defining feature of PCOS, manifesting in clinical symptoms such as hirsutism, acne, and androgenic alopecia. Elevated DHT levels drive hirsutism by stimulating hair follicle growth in areas like the upper lip, chin, and lower abdomen. Likewise, androgen-induced sebaceous gland hyperplasia leads to increased sebum production, contributing to acne, while the conversion of terminal hair follicles into vellus hair results in androgenic alopecia [73]. Beyond these cosmetic concerns, hyperandrogenism disrupts the HPO axis. Elevated androgens alter GnRH pulsatility, creating an imbalance between FSH and LH, which impairs follicular development, triggers anovulation, and ultimately contributes to infertility. Furthermore, hyperandrogenism can negatively affect endometrial physiology—raising the risk of hyperplasia and cancer—and lead to menstrual irregularities such as oligomenorrhea, amenorrhea, and irregular uterine bleeding.

ROS play a crucial role in modulating androgen synthesis and metabolism by influencing the activity and expression of key enzymes. ROS can enhance the activity of CYP17A1, a rate-limiting enzyme that converts pregnenolone and progesterone into androstenedione and dehydroepiandrosterone (DHEA), through pathways such as MAPK signaling [74,75]. In addition, ROS have been implicated in altering the activities of 3β-hydroxysteroid dehydrogenase (3β-HSD) and 17β-hydroxysteroid dehydrogenase (17β-HSD), enzymes responsible for converting DHEA into androstenedione and then testosterone, respectively. Moreover, ROS influence androgen metabolism by inhibiting sulfotransferase (SULT) enzymes, which normally sulfate and inactivate androgens, thereby increasing the levels of bioactive androgens. They also affect the synthesis and secretion of SHBG in the liver; reduced SHBG levels result in higher concentrations of free, active androgens in circulation [76]. Collectively, these ROS-mediated alterations in enzyme activity and hormone binding contribute to the elevated androgen levels observed in PCOS, reinforcing the interplay between oxidative stress and hyperandrogenism in the disorder. The details of the relationship between ROS and hyperandrogenism are summarized in Fig. 2.

### 3.4 Relationship between ROS and obesity

Obese PCOS patients exhibit distinct clinical and metabolic profiles compared to their non-obese counterparts. Clinically, they display typical PCOS symptoms—such as menstrual irregularities, hirsutism, and acne—which are often more severe due to obesity. These patients generally have a BMI ≥25 kg/m^2^ and commonly present with abdominal obesity (waist-to-hip ratio >0.85), a condition closely linked to metabolic complications. Obesity exacerbates hyperandrogenism and insulin resistance, with obese patients frequently experiencing denser, more extensive hirsutism and more persistent acne [77]. Metabolically, these individuals exhibit higher fasting and postprandial insulin levels, elevated indices of insulin resistance, and greater disturbances in glucose and lipid metabolism—factors that increase their risk of developing type 2 diabetes (3–5 times higher than non-obese PCOS patients) and cardiovascular diseases [78].

Obesity contributes to PCOS pathogenesis through multiple interrelated mechanisms. Chronic inflammation is a key pathway; hypertrophic adipocytes secrete pro-inflammatory cytokines such as TNF-α, IL-6, and IL-1β, which disrupt the HPO axis by altering GnRH pulsatility, increasing the LH/FSH ratio, and impairing follicular development. These inflammatory mediators also suppress hepatic production of SHBG, thereby elevating free androgen levels and worsening hyperandrogenism [2,18]. Furthermore, obesity alters adipokine secretion—marked by increased leptin levels (leading to leptin resistance) and decreased adiponectin levels—thereby exacerbating insulin resistance and metabolic dysfunction. In addition, obese adipose tissue produces excessive ROS, which intensify oxidative stress [79]. Elevated ROS damages ovarian tissue by interfering with FSH and LH signaling, inhibiting GC proliferation and differentiation, and promoting apoptosis. This oxidative stress further impairs insulin receptor substrate signaling through serine phosphorylation, thereby deepening insulin resistance and perpetuating the metabolic disturbances characteristic of PCOS [80,81]. The details of the relationship between ROS and obesity are summarized in Fig. 2.

### 3.5 Relationship between ROS and insulin resistance

Insulin resistance is a core pathogenic factor in PCOS, affecting up to 70–80 % of patients. It reduces the body's ability to effectively utilize insulin, resulting in elevated blood glucose levels and compensatory hyperinsulinemia. This hyperinsulinemia not only worsens insulin resistance but also contributes to metabolic disturbances, including dyslipidemia and an increased cardiovascular risk. PCOS patients typically exhibit higher fasting and postprandial blood glucose levels, as well as elevated glycated hemoglobin, which correlate with the severity of insulin resistance [82].

Furthermore, insulin resistance disrupts lipid metabolism. Obese PCOS patients often display dyslipidemia—characterized by elevated triglycerides, increased LDL-C, and reduced HDL-C—driven by enhanced free fatty acid release from adipocytes, which impairs insulin signaling and promotes hepatic synthesis of triglycerides and very low-density lipoprotein [83]. These metabolic derangements further elevate cardiovascular risk [84]. Additionally, hyperinsulinemia stimulates the hypothalamus to increase GnRH secretion, leading to an imbalanced LH/FSH ratio that disrupts follicular development and ovulation, while simultaneously suppressing hepatic production of SHBG. This results in higher free androgen levels, exacerbating hyperandrogenism [10,85].

ROS disrupt insulin signaling by activating serine kinases—such as c-Jun N-terminal Kinase (JNK), NF-κB-inducing kinase, and IKK—which phosphorylate serine residues on the insulin receptor substrate, thereby impeding its tyrosine phosphorylation and downstream signaling [86]. In parallel, ROS induce oxidative modifications in key components of the insulin signaling cascade, for example impairing the interaction between the regulatory subunit p85 and the catalytic subunit p110 of PI3K, which diminishes Akt (Protein Kinase B) activation and GLUT4 translocation. Mitochondrial dysfunction—characterized by reduced respiratory chain activity, diminished ATP production, and increased ROS generation—further compounds these effects, with mtDNA deletions and elevated plasma GDF-15 levels being linked to insulin resistance in PCOS [87]. The severity of oxidative stress directly correlates with the degree of insulin resistance in PCOS patients [88,89].

In addition, ROS foster a pro-inflammatory state by activating the NF-κB pathway, thereby boosting the production of cytokines such as TNF-α and IL-6. These inflammatory mediators further compromise insulin signaling; TNF-α enhances JNK activity leading to additional inhibitory serine phosphorylation of insulin receptor substrate, while IL-6 diminishes SHBG synthesis, resulting in higher levels of free androgens. Chronic inflammation in adipose tissue thus exacerbates insulin resistance and the metabolic abnormalities observed in PCOS [62,90]. The details of the relationship between ROS and insulin resistance are summarized in Fig. 2.

### 3.6 Systemic effects and pathway driven by ROS

Beyond the ovary, ROS-induced oxidative stress in PCOS contributes to systemic metabolic disturbances and inflammation. Elevated ROS levels in skeletal muscles are linked to insulin resistance, with antioxidant interventions such as NAC showing improvements in glucose uptake and metabolic profiles [91]. Hepatic mitochondrial dysfunction accompanied by increased ROS has been implicated in early liver fibrosis, suggesting that oxidative stress underlies broader metabolic syndrome features [92]. Inflammatory pathways are also activated by ROS, with upregulation of NF-κB, TNF-α, and IL-6 exacerbating both local and systemic inflammation [53,93]. Novel therapies—including sulforaphane, curcumin, and electroacupuncture—have demonstrated the ability to suppress these inflammatory responses by modulating pathways such as AMPK/AKT/NRF2 and NF-κB, thereby restoring ovarian function [8,94,95]. Emerging gene regulatory approaches, involving microRNAs (e.g., miR-93-5p, miR-196b-5p) and RNA-binding proteins (e.g., YTHDF2), further offer promising strategies to attenuate ROS-induced damage and improve mitochondrial integrity [49,50,96].

The comprehensive detailing of ROS's impact across follicular development, hyperandrogenism, obesity, and insulin resistance reveals ROS not merely as a biomarker of stress, but as a central pathogenic hub that actively drives and integrates the diverse clinical manifestations of PCOS. This emphasizes that targeting ROS is not just about reducing oxidative damage but about disrupting a core mechanism of disease progression. The strong correlations between specific ROS biomarkers and clinical outcomes, such as oocyte quality and in vitro fertilization success, suggest that routine monitoring of these markers could provide a more dynamic and personalized approach to PCOS management, enabling earlier diagnosis, more precise risk stratification, and real-time assessment of treatment efficacy.

Table 2 provides an integrative overview of the diverse molecular mechanisms by which ROS contribute to the pathogenesis of PCOS. The table illustrates that in PCOS, ROS-mediated cellular damage is not attributable to a single pathway but rather results from a complex interplay of processes. For example, in KGN cells, DHEA or homocysteine (Hcy) exposure induces ferroptosis via enhanced lipid peroxidation and GSH depletion [64,97]. Mitochondrial dysfunction is another central feature, where NAD^+^ deficiency in human GCs and PCOS mice leads to ROS accumulation and reduced ATP production [28,42]. Additionally, elevated levels of follicular fatty acids, such as oleic acid, trigger inflammasome activation via ERK1/2 and NLRP3 pathways in KGN cells and PCOS rats [98,99].

**Table 2:** Mechanisms linking ROS to PCOS pathogenesis.

Mechanisms	Key ROS-Related Findings	Ref
AKT inactivation	ROS suppresses AKT → glycolytic dysfunction	[100]
↓Antioxidant	↓SOD, ↓Catalase, ↓GSH activity; ↑MDA/8-OHdG in FF	[52,59]
Apoptosis	Activated estrogen receptor trigger FOXO1-ROS–NF–κB axis; Imbalanced Drp1 phosphorylation → mitochondrial fission	[45,56,101]
↑Autophagy	ROS/PI3K/AKT/mTOR imbalance → excessive autophagy in GCs	[102]
Ferroptosis	DHEA/Hcy → ferroptosis with ↑ROS, lipid peroxidation, and ↓GSH	[64,97]
Fibrosis	p66Shc promotes ROS-driven fibrosis	[54]
Genetic variants	SOD gene polymorphisms impair ROS elimination in PCOS women	[103]
Inflammasome activation	Activation of ERK1/2 and NLRP3 pathways	[98,99]
Insulin resistance	Mitochondrial SIRT3↓ → glucose metabolism defects	[104]
Mitochondrial dysfunction	↑ROS, ↓ATP, ↓NAD^+^, ↓HIF-1α	[28,42,105]
Mitophagy defect	HIF-1α/BNIP3 axis disruption impairs mitochondrial quality control	[106]
Oxidative stress	↑Aminolevulinate Synthase 2 (Alas2) → oxidative damage in GCs	[94]
Proteostasis defect	Insulin resistance disrupts mitochondrial proteostasis via UPR^mt^ defects	[29]
Steroid disruptions	SPRY4 knockdown → ↓ROS and improves steroidogenesis	[107]
Zinc deficiency	Impairs mitochondrial dynamics → ↑ROS, SOD2 acetylation	[30]

Furthermore, Table 2 highlights that ER stress and apoptosis, triggered by factors like linoleic acid and DHT through the ER-FOXO1-ROS–NF–κB axis in KGN cells and zebrafish, contribute to GC loss [45,101]. Impaired antioxidant defenses are evident from reduced activities of enzymes such as SOD, Catalase, and GSH, with concomitant increases in markers like MDA/8-OHdG in the FF of PCOS patients [52,59]. Moreover, mitochondrial SIRT3 dysfunction connects oxidative stress to insulin resistance, as seen in both PCOS mice and human GCs [59,104]. Other mechanistic insights include excessive autophagy driven by an imbalance in the ROS/PI3K/AKT/mTOR axis [102], genetic variations (e.g., SOD gene polymorphisms) that impair ROS elimination [103], and additional pathways such as mitophagy dysregulation [106], AKT pathway inactivation [100], and mt-lysosome axis failure [29]. The table also details novel mechanisms, including the roles of Alas2 overexpression [94], SPRY4-ERK1/2 phosphorylation [107], and the interplay between p66Shc-Sirt1 [54], the ADMA-DDAH1 axis [56], and Hypoxia-Inducible Factor 1-alpha (HIF-1α) signaling [105]. Furthermore, mechanisms involving TFRC-mediated iron uptake, miR-196b-5p regulation, the YTHDF2-MSS51 axis, and zinc deficiency underscore the multifaceted contribution of oxidative stress to PCOS pathogenesis [30,49,50,60].

## 4 Experimental models and therapeutic interventions in ROS-mediated PCOS

### 4.1 Experimental models for studying ROS in PCOS

To study the role of ROS in PCOS pathogenesis, a comprehensive model induction method is needed. Table 3 presents a comprehensive overview of various cell and animal models employed to investigate the role of ROS in the pathogenesis of PCOS. These models simulate key ROS-related phenotypes—such as lipid peroxidation, mitochondrial dysfunction, apoptosis, and inflammation—allowing researchers to evaluate the efficacy of different interventions. For instance, DHEA-treated KGN cells reveal hyperandrogen-induced ferroptosis, increased ROS, and lipid peroxidation, which can be mitigated by agents like 1,25-Dihydroxyvitamin D3, atractylodin, and Nuciferine (NF) [64,108,109]. Similarly, Letrozole combined with a high-fat diet (HFD) in PCOS rats leads to elevated levels of MDA, ROS, and inflammatory cytokines.

**Table 3:** In vitro/animal models for ROS studies in PCOS.

Model	Induction	Key ROS-Related Phenotype	Ref
KGN cells	DHEA (10 μM) 24h	↑ROS, and lipid peroxidation	[64,108,109]
KGN cells	DHT (25 nM) 48h, DHT (500 nM) 24h	Glycolytic failure and apoptosis	[100,110]
KGN cells	Palmitic acid (100 μM) 24h	ROS accumulation and insulin resistance	[29]
KGN cells	BPA (100 μM) 24h	Mitochondrial dysfunction, apoptosis	[111]
KGN cells	LPS (1 μg/mL) 24h	↓NAD^+^, inflammation	[42]
SVOG cells	DHT (10/100 nM) 48h	↑ROS and ↓ΔΨm	[45]
Granulosa-lutein cells	From PCOS patients	↑ROS and apoptosis	[95,112]
Zebrafish; CHO cells	BPA (10 μg/L) 15d; BPA (4.4 μM) 24h	Mitochondrial ROS, apoptosis	[113]
Mouse GCs	LPS (10 μg/mL) 24h	ROS-dependent apoptosis and inflammation	[114]
Porcine oocytes	TPEN (4 μM) 24h	Zinc deficiency → ↑ROS, mitophagy, apoptosis	[30]
Rats	Letrozole (2 mg/kg) + HFD (60 %) 7w	ROS-driven apoptosis	[115]
Rats	Letrozole (1 mg/kg) 4w + HFD (40 %) 16w	↑MDA, ↑ROS, and inflammatory cytokines	[52]
Rats	T (0.1 mg/0.004 ml olive oil per g of animal) 9d-8w	Oxidative stress, ↑blood glucose, disordered estrous cycle	[104]
Rats	DHEA (6 mg/100g) 35d	Oxidative stress, inflammation; ↑ROS and fibrosis	[54,99]
Rats	DHEA (6 mg/100g) 20/21d	↑ROS, ↑MDA, ↑IL-6	[55,116]
Rats	DHEA (8 mg/100g) 25d	↑Leukocyte ROS levels	[117]
Mice	DHEA (6 mg/100g) 20d/DHEA + HFD 21d	↓Antioxidant enzymes, ↑ROS in oocytes	[8,70]
Mice	DHT (250 μg) on 16.5, 17.5 and 18.5 days post coitus	Oocyte ROS accumulation due to mitochondrial dysfunction	[118]
Hamsters	Letrozole (3 mg/kg)/Melatonin (1 mg/kg) 40 days	Uterine oxidative stress, metabolic dysfunction	[119]

Moreover, Table 3 highlights the utility of diverse models—from BPA-exposed zebrafish and CHO cells, which show mitochondrial ROS and apoptosis reversible by luteolin and 1,25-Dihydroxyvitamin D3 [111,113], to DHT-induced GC injury models where ROS-mediated mitochondrial fission and apoptosis are counteracted by compounds like carnosol, berberine, and growth hormone [45,110,112]. Other models, such as LPS-treated embryos/GCs and cold-exposed PCOS rats, further underscore the multifaceted mechanisms of ROS-induced damage and the range of interventions tested, including berberine, NMN (Nicotinamide Mononucleotide), and controlled cold exposure [114,117,120]. Additional models address metabolic disruptions, such as palmitic acid-treated GCs showing mt/mitophagy defects, and prenatal androgen-exposed mice with oocyte ROS accumulation, where antioxidant therapies (e.g., Auraptene) are explored [29,118,121]. Notably, models examining brain structure alterations in DHEA-induced PCOS rats and skeletal muscle cell oxidative stress also offer insights into the systemic impact of ROS and potential therapeutic avenues [8,55,70,116]. Collectively, these in vitro and animal models provide critical insights into the molecular mechanisms by which oxidative stress contributes to PCOS pathology, while also identifying potential targets for therapeutic intervention.

### 4.2 Therapeutic strategies targeting ROS in PCOS

#### 4.2.1 Signaling pathways and therapeutic modulation of ROS

The detrimental effects of ROS extend to various intracellular signaling pathways that govern cell survival, proliferation, and metabolism. For instance, growth hormone activates the PI3K/Akt pathway, which has been shown to reduce ROS accumulation and apoptosis in GCs [112]. Epigenetic regulators such as Histone Deacetylase 5 and metabolic agents like liraglutide enhance the activity of antioxidant defenses, thereby mitigating oxidative stress [122,123]. Metformin, a widely used therapeutic in PCOS, has been repeatedly demonstrated to remodel mitochondrial morphology and suppress ROS production via mTOR-dependent and miRNA-mediated pathways (e.g., miR-670-3p/NOX2) [102,123,124]. Additionally, treatments with 1,25-dihydroxyvitamin D_3_ and N-acetylcysteine (NAC) restore mitochondrial membrane potential and counteract chemical-induced ROS elevation, highlighting the promise of targeted antioxidant therapies [111,125].

#### 4.2.2 Common techniques and indicators for detecting ROS levels

A variety of techniques have been developed to quantify ROS in clinical samples, each with distinct advantages and limitations. The fluorescent probe method, particularly using DCFH-DA (2′,7′-dichlorodihydrofluorescein diacetate), is widely employed for intracellular ROS detection. DCFH-DA is non-fluorescent and diffuses freely into cells, where intracellular esterases hydrolyze it to DCFH. Upon oxidation by ROS, DCFH is converted to the highly fluorescent compound DCF, with fluorescence intensity—measured via fluorescence microscopy, flow cytometry, or confocal microscopy—directly correlating with ROS levels. For example, studies on ovarian GCs from PCOS patients have demonstrated significantly higher fluorescence intensity compared to controls, indicative of elevated ROS levels [5]. Similarly, the chemiluminescence method employs luminol, which, when oxidized by ROS, emits photons; this technique is highly sensitive and has revealed increased serum chemiluminescence in PCOS patients [126].

Other approaches include electron paramagnetic resonance spectroscopy, which directly detects free radicals with unpaired electrons (such as superoxide and ·OH) by measuring their magnetic resonance signals, though its application is limited by costly instrumentation and complex sample preparation [127]. Spectrophotometric methods, which rely on detecting characteristic light absorption of ROS-reactive products, offer a simpler and more cost-effective alternative, albeit with lower sensitivity and potential interference from other substances. Common ROS indicators include O_2_^−^, H_2_O_2_, and ·OH. Superoxide can be measured using the cytochrome *c* reduction assay, H_2_O_2_ levels are often inferred via catalase activity, and ·OH can be detected using high-performance liquid chromatography or fluorescence-based assays [5,128].

#### 4.2.3 The value of ROS level detection in the diagnosis and disease assessment of PCOS

Detecting ROS levels holds significant promise for the early diagnosis of PCOS, disease progression monitoring, and treatment efficacy assessment. In the initial stages of the disorder, patients may present with only subtle metabolic and endocrine disturbances, and traditional diagnostic markers, such as sex hormone profiles and ultrasound imaging—may appear normal. However, emerging evidence suggests that elevated ROS levels occur early in the disease course. For example, a prospective study tracking adolescent girls for 2–3 years post-menarche found that those who later developed PCOS exhibited higher serum ROS levels compared to controls, indicating that ROS detection may help identify high-risk individuals [129].

Furthermore, regular monitoring of ROS levels can provide valuable insights into disease progression and treatment efficacy. As PCOS advances, oxidative stress intensifies, particularly in obese patients, correlating with worsening insulin resistance and metabolic abnormalities. Rising ROS levels during therapy may indicate suboptimal treatment response, thereby guiding clinicians to adjust interventions—such as enhanced antioxidant regimens or intensified lifestyle modifications—to better control the condition. Additionally, since elevated ROS is linked to long-term complications like type 2 diabetes and cardiovascular diseases, routine ROS monitoring can aid in risk stratification and prompt early preventive measures [130].

### 4.3 PCOS treatment strategies targeting the regulation of ROS

#### 4.3.1 Applied research of antioxidants in the treatment of PCOS

**Fig. 3:**
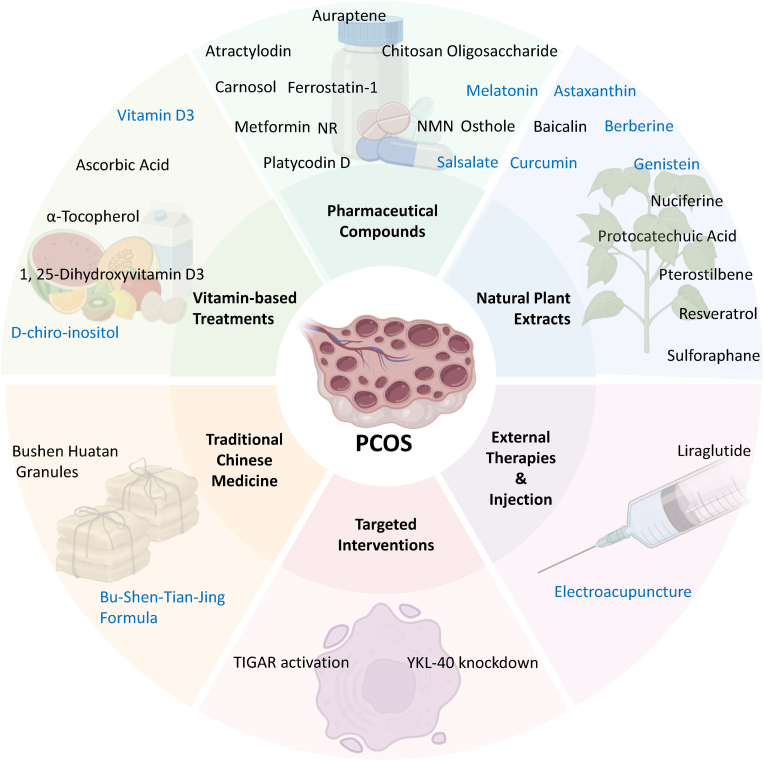
Emerging Therapeutic Strategies for PCOS Targeting Oxidative Stress-Related Dysregulation. This figure illustrates recent advances in PCOS treatment, categorized into six interconnected therapeutic approaches. Those clinically applied are highlighted in blue.

Antioxidants hold potential in treating PCOS by mitigating oxidative stress. Vitamin C, a water-soluble antioxidant, scavenges ROS and maintains cellular redox balance. Clinical studies show that 1000 mg/day of vitamin C for 8 weeks improves insulin resistance, reduces blood glucose levels, and enhances antioxidant enzyme activities in PCOS patients. Vitamin E, a fat-soluble antioxidant, protects cell membranes from lipid peroxidation. A 12-week trial with 400 IU/day of vitamin E reduced serum testosterone levels and improved hyperandrogenism symptoms [131]. The details of PCOS treatment strategies are summarized in Fig. 3.

NAC enhances GSH levels, improving insulin sensitivity and ovarian function. A dose of 1.2–1.8 g/day for 1–6 months is effective in enhancing ovulation rates, especially in clomiphene-resistant patients. Melatonin, with its antioxidant and circadian rhythm-regulating properties, improves ovarian morphology and function in PCOS models by reducing oxidative stress and regulating the HPO axis [132]. Natural plant extracts like resveratrol and baicalin also show promise. Resveratrol activates SIRT1, reducing ROS levels and improving ovarian function in PCOS models. Baicalin alleviates ovarian pathology in PCOS models by enhancing antioxidant enzyme levels and mitochondrial function. These findings suggest that antioxidants can be valuable adjuncts in managing PCOS [62].

#### 4.3.2 Experimental therapeutic interventions targeting ROS in PCOS

A diverse array of therapeutic strategies aims to mitigate oxidative stress in PCOS by targeting ROS through multiple mechanistic pathways. These interventions span from classical antioxidants and vitamin metabolites to herbal extracts and pharmaceutical agents, each acting via specific molecular targets to restore redox balance and improve ovarian function. The extensive list of promising interventions from in vitro and animal models often contrasts with the relatively fewer robust human clinical trials that demonstrate significant reproductive outcomes (Fig. 3). This highlights a critical translational gap in PCOS research, where preclinical success does not consistently translate to clinical efficacy, underscoring the need for more clinical validation.

Table 4 outlines a diverse array of therapeutic strategies aimed at mitigating oxidative stress in PCOS by targeting ROS through multiple mechanistic pathways. These interventions span from classical antioxidants and vitamin metabolites to herbal extracts and pharmaceutical agents, each acting via specific molecular targets to restore redox balance and improve ovarian function. For instance, 1,25-dihydroxyvitamin D3 has been shown to reduce ROS, lipid peroxidation, and ferroptosis in DHEA-treated KGN cells, thereby alleviating hyperandrogen-induced oxidative damage [64]. Similarly, the combination of ascorbic acid (AA) and α-tocopherol enhances total antioxidant capacity (TAC), decreases MDA levels, and upregulates key antioxidant enzymes (Catalase, SOD, HO-1), resulting in improved oxidative status and ovarian function in PCOS mice [133].

**Table 4:** Therapeutic interventions targeting ROS in PCOS within animal and cell models.

Intervention	Mechanism/Effect	Outcome	Ref
1,25-Dihydroxyvitamin D3	Reduces ROS, lipid peroxidation, and ferroptosis in DHEA-treated KGN cells	Alleviates hyperandrogen-induced oxidative damage	[64]
Ascorbic Acid & α-Tocopherol	↑TAC, ↓MDA, upregulates antioxidant enzymes (Catalase, SOD, HO-1)	Improves ovarian function in PCOS mice	[133]
Astaxanthin	↓Oxidative stress and apoptosis	Protects GCs in PCOS mice	[134]
Astaxanthin + Curcumin	↓ROS, ↓MDA, ↑Catalase, ↓IFN-γ, ↓IL-6	↓ Inflammation, ↑ovarian function in PCOS mice	[8]
Atractylodin	Inhibits ferroptosis via PDK4-mediated JAK-STAT3 pathway; ↓ROS, ↓MDA	Protects GCs from DHEA-induced damage	[108]
Auraptene	Enhances GSH synthesis; reduces ROS in oocytes	Improves oocyte maturation and mitochondrial function	[69]
Berberine	Suppresses ROS/caspase-3-dependent apoptosis and NF-κB signaling	Mitigates embryo damage and DHT-induced GC injury	[51,120]
Bushen Huatan Granules	Reduces mitochondrial ROS	Ameliorates DHEA-induced PCOS in rats	[116]
Carnosol	↑Nrf2/HO-1 via Keap1 inhibition; reverses ROS and apoptosis	Attenuates PCOS phenotypes in mice	[110]
Chinese Herbal Medicine (BSTJF)	↑mitochondrial SIRT3 signaling; improves glucose metabolism and OS	Alleviates PCOS pathogenesis	[104]
Chitosan Oligosaccharide	↓ ROS via HIF-1α/VEGFA suppression; ↓ inflammatory cytokines	Improves GC proliferation and reduces apoptosis	[135]
Curcumin	↑PPAR-γ, ↓ROS, ↓MDA, ↑SOD, ↑GPx, ↑GSH	Improves ovarian function in PCOS rats	[136]
Electroacupuncture	Downregulates Alas2; reduces oxidative stress in GCs	Improves ovarian function in PCOS rats	[94]
Ferrostatin-1	↓TET-mediated DNA methylation; ↓apoptosis and oxidative stress	Rescues Hcy-induced GC ferroptosis	[97]
Genistein	↑ ER-Nrf2-Foxo1-ROS pathway; enhances antioxidant defenses	Ameliorates PCOS symptoms in mice	[137]
Liraglutide	Reduces ovarian ROS levels; synergizes with metformin	Improves PCOS symptoms in rats	[138]
Metformin	Suppresses miR-670-3p/NOX2/ROS pathway; inhibits pyroptosis and OS	Reduces GC apoptosis and oxidative stress	[124]
Melatonin	↓ROS, ↑Gpx1/Sod1, ↑Bcl2/Bax ratio	↑Oocyte maturation (mice)	[70]
Melatonin	↓Oxidative/inflammatory	↑Uterine function (hamsters)	[119]
Nicotinamide Mononucleotide	Restores NAD^+^ levels; inhibits TLR4/NF-κB/MAPK pathways	Reduces ROS and improves oocyte quality	[114]
Nicotinamide Riboside (NR)	Restores NAD^+^ levels; reduces ROS and mitochondrial dysfunction	Improves ovarian function in PCOS mice	[28]
Nuciferine (NF)	↓ferroptosis via SOX2/SLC7A11/GPX4; ↓ROS, ↓MDA	Protects DHEA-treated KGN cells	[109]
Osthole	↑Nrf2-Foxo1-GSH axis; ↓NF-κB-mediated inflammation	↓Oxidative stress and improves folliculogenesis	[139]
Platycodin D	Inhibits ferroptosis via CD44 modulation; reduces lipid peroxidation	Protects ovarian tissue in PCOS rats	[48]
Protocatechuic Acid	Activates PI3K signaling; reduces GC apoptosis and ROS	Restores ovarian antioxidant defenses	[46]
Sulforaphane	Activates AMPK/AKT/NRF2 pathway	Reduces ROS and apoptosis in GCs of PCOS patients	[95]
TIGAR activation	Activates Nrf2, ↓MDA, ↓ROS, ↑GPx, ↑SOD	Inhibits GC apoptosis and oxidative stress in PCOS rats	[65]
Vitamin D3	↑Mitochondrial biogenesis via MAPK pathway; ↓ROS	↑ Mitochondrial function in GCs of PCOS mice	[140]
YKL-40 knockdown	↑Antioxidant capacity, ↓apoptosis, ↓inflammation	Protects GCs from H_2_O_2_-induced oxidative damage	[61]

Additional interventions such as astaxanthin, atractylodin, and berberine modulate pro-inflammatory cytokines, ferroptosis, and apoptosis through activation of protective pathways like Nrf2/HO-1 or inhibition of NF-κB signaling, contributing to reduced oxidative injury in both GCs and whole animal models [51,108,120,141]. Other promising agents include natural compounds (e.g., genistein, auraptene, osthole, sulforaphane) and strategies that restore NAD^+^ levels (NMN, NR), which collectively target mitochondrial dysfunction, ER stress, and insulin resistance—key features of PCOS-related oxidative stress [28,114,124,137,138]. Complementary approaches, such as electroacupuncture and Chinese herbal medicine (BSTJF), further demonstrate the potential to downregulate ROS production and improve metabolic as well as reproductive outcomes [94,104,116]. Moreover, innovative interventions like YKL-40 knockdown and treatment with Chitosan Oligosaccharide have been shown to enhance cellular antioxidant defenses and reduce apoptosis, underscoring the multifaceted nature of ROS-targeted therapies [61,135].

Collectively, these findings highlight the therapeutic promise of targeting ROS in PCOS, as interventions not only diminish oxidative stress markers but also translate into improved cellular function and overall reproductive outcomes. This comprehensive strategy offers multiple points of intervention that may be tailored to address the complex interplay between oxidative stress and PCOS pathogenesis.

#### 4.3.3 Clinical therapeutic interventions targeting ROS in PCOS

Table 5, provides a concise summary of key therapeutic interventions identified in human clinical studies that address ROS-related mechanisms in PCOS and their reported reproductive outcomes. This structured presentation is invaluable for clinicians and researchers, enabling a rapid assessment of current evidence, identification of promising treatment avenues, and recognition of areas requiring further investigation. The inclusion criteria for this table were strictly limited to interventions with reported human clinical data pertaining to both their ROS-related mechanisms (or strong links to pathways known to influence ROS in humans) and their impact on reproductive outcomes.

**Table 5:** Key therapeutic interventions in human PCOS targeting ROS and their reproductive outcomes.

Intervention	Mechanism/Effect	Outcome	Ref
Astaxanthin	Reductions in oxidative stress and inflammatory cytokines (IL-1β, IL-6).	Significantly improved number of oocytes retrieved, MII oocyte count, oocyte maturity rate, and number of frozen embryos in ART patients.	[141]
Berberine Phytosome (BP)	Alleviates insulin resistance, reduces serum androgen levels, regulates lipid metabolism, and mild chronic inflammation.	Resumption of regular menstruation (approx. 70 % of women), normalization of ovarian anatomy (over 60 % of women). May improve fertility and pregnancy outcomes.	[142]
Chinese Herbal Medicine (e.g., BSTJF)	Reduces ROS, improves glucose metabolism and inflammation. Alters serum hormones to normal levels.	More retrieved oocytes, more fertilized oocytes, significantly higher clinical cumulative pregnancy rate, live birth rate, and term delivery rate.	[143]
Curcumin	Potent antioxidant and anti-inflammatory properties reduce oxidative stress and related metabolic complications.	Reduces the risk of abnormalities of glucose and lipid metabolism and obesity in patients with PCOS	[144]
D-chiro-inositol	Reduction in oxidative stress, increase in antioxidant enzyme (CAT, SOD) activities.	Improved metabolic profile, and hormonal profiles with reduced testosterone and improved ovarian function.	[121]
Electroacupuncture	Decreases serum ROS and insulin resistance, increases SOD.	Improves menstrual cycle, serum hormone (LH, FSH, E2, A4, T) and ovulation.	[145]
Isoflavone (Genistein)	Reduces oxidative stress and inflammation by increasing antioxidative levels.	Lowering testosterone level, normalizing menstrual cycle, and restoring normal ovarian morphology.	[146]
Melatonin	Increasing the expression of antioxidant enzymes.	Improves oocyte quality, increases potential for successful fertilization, improves pregnancy outcomes.	[147]
Salsalate (Salicylate)	Suppresses ROS generation, activated NF-κB, and circulating tumor necrosis factor-α.	Normalized basal androgen levels, lowered HCG-stimulated androgen secretion, induced ovulation in 4 out of 8 previously anovulatory women.	[53]
Vitamin D3	Regulates sex hormones and affects reproductive tissues via Vitamin D Receptor and 1α-hydroxylase activity.	Improved ovarian morphology, regularity of menstrual cycles, significant increase in ovulation rate. Reduction in mean testosterone levels.	[148]

A notable distinction emerges between interventions that directly measure and modulate ROS markers and those whose ROS-related benefits are more indirectly inferred. Treatments like Electroacupuncture and Salsalate have demonstrated direct effects on reducing serum ROS levels or suppressing ROS generation in human patients [53,145]. This direct modulation of oxidative stress is a clear therapeutic pathway. In contrast, for agents such as Berberine [142], while their positive impact on reproductive outcomes is well-documented, the explicit measurement of ROS reduction in human clinical trials is less consistently reported in the provided literature. Their efficacy is often attributed to improvements in insulin sensitivity or inflammation, which are known to contribute to oxidative stress in PCOS. This distinction is important for understanding the primary mechanisms of action and for designing more targeted interventions in the future.

Despite the promising findings, several limitations in the current human clinical literature warrant consideration. For many interventions, the direct measurement of ROS reduction in human clinical trials is not always explicitly detailed, even when the intervention possesses known antioxidant properties or affects pathways linked to ROS. This represents a significant gap in our understanding of the precise ROS-modulating effects in human patients. Furthermore, while animal and cell line studies provide valuable mechanistic insights, their findings cannot be directly extrapolated to human physiology, underscoring the critical need for more human-specific mechanistic research. The variability in how “reproductive outcomes” are defined and measured across studies—ranging from improved ovulation rates and oocyte quality to live birth rates—also poses challenges for direct comparisons and comprehensive meta-analyses. Additionally, some of the studies are characterized by small sample sizes or retrospective designs, which can limit the generalizability of their findings.

## 5 Discussions

Recent studies show that in women with PCOS, total oxidant status and ROS levels are significantly elevated in serum and follicular fluid, while antioxidant defenses are diminished, indicating a marked imbalance in oxidative status [149]. Our systematic review of original research (2020–2025) confirmed these findings: PCOS is characterized by an oxidant–antioxidant imbalance, with oxidative biomarkers elevated by roughly 30 % compared to controls and enzyme activities (SOD, GPx) often suppressed [103,109]. This pervasive oxidative stress correlates with clinical severity and fertility outcomes, suggesting it is a core feature – not a mere bystander – of PCOS pathology.

Given these multifaceted interactions, understanding the molecular interplay between ROS and PCOS is critical. Mitochondrial dysfunction (e.g. impaired complex I/III activity or mtDNA mutations) increases ROS production and reduces ATP output, which in turn deranges insulin signaling [20]. Elevated ROS activate stress kinases (JNK, p38) and the NF-κB pathway, promoting serine phosphorylation of insulin receptor substrates and effectively blocking normal insulin signaling [78,150]. The result is tissue-wide insulin resistance: insulin-stimulated glucose uptake is blunted, leading to compensatory hyperinsulinemia that further feeds ROS production (a vicious cycle). In parallel, ROS influence ovarian steroidogenesis. Oxidative stress–activated pathways (e.g. ROS/p38-MAPK, JNK) can upregulate key enzymes such as CYP17A1 and CYP19A1, skewing steroidogenesis toward androgen excess. For instance, in PCOS theca cells, saturated fat–induced ROS activated JNK/p38 signaling to increase androgen output and promote ovarian fibrosis [57]. Conversely, hyperandrogenism itself amplifies ROS: excess androgens sensitize leukocytes and GCs to glucose, boost NOX4 activity, and trigger endoplasmic reticulum stress pathways that raise ROS levels. In vitro studies even show ROS can suppress HNF-4α and lower SHBG, raising free testosterone. Thus, ROS and hyperandrogenism form an interlinked feedback loop that aggravates insulin resistance and hormonal imbalance.

Obesity and chronic inflammation further amplify oxidative stress in PCOS. Adiposity increases free fatty acids and pro-inflammatory cytokines, which stimulate ROS production and impair mitochondrial quality. In overweight/obese PCOS patients, weight loss rapidly lowers leukocyte ROS output and lipid peroxidation, underscoring how excess weight directly fuels oxidative stress. Independent of obesity, PCOS is marked by low-grade inflammation: circulating levels of TNF-α, IL-6, IL-8 and C-Reactive Protein are elevated relative to controls. These cytokines activate toll-like receptors and NF-κB in ovarian and metabolic tissues, triggering NLRP3 inflammasome assembly and further ROS release. Inflammation also downregulates SHBG production and perturbs steroidogenesis, raising free androgens [76]. Collectively, adipokine imbalances and inflammation in PCOS patients set up a feed-forward loop: inflammatory mediators generate ROS (via NADPH oxidases and mitochondrial damage) which then promote additional insulin resistance and hyperandrogenism, while ROS in turn reinforce inflammatory signaling. Thus, obesity and chronic inflammation act in concert with ROS, linking the metabolic and reproductive facets of PCOS.

Given this ROS-centric view of PCOS pathogenesis, antioxidant strategies have been widely explored as adjunctive treatments. A broad array of agents has shown promise in preclinical and early clinical studies: Vitamins C and E, α-lipoic acid, selenium and GSH precursors (e.g. NAC) have been trialed. These generally improve oxidative stress biomarkers (lower MDA, raise TAC), enhance insulin sensitivity and sometimes restore ovulation [151]. For example, NAC has improved menstrual cyclicity and fertility outcomes in clomiphene-resistant PCOS subjects. Natural compounds such as resveratrol and melatonin exhibit both antioxidant and metabolic benefits. Resveratrol activates SIRT1 and inhibits the pro-oxidant protein p66Shc, preventing insulin resistance and ovarian oxidative damage in animal PCOS models. Melatonin (secreted in the follicle) directly scavenges ROS and upregulates intracellular antioxidants; supplementation has been reported to improve oocyte quality and pregnancy rates in assisted reproduction. Traditional Chinese medicine (e.g. compounds containing flavonoids, polyphenols and vitamins) and inositol isomers also counter oxidative stress and support ovarian function [8,70]. Clinical trials of myo-inositol and D-chiro-inositol, often in combination with antioxidants, consistently show improved insulin sensitivity, reduced androgens and better menstrual regularity. These data suggest antioxidants can enhance the efficacy of standard treatments by targeting the oxidative component of PCOS. Hence, antioxidant therapy is viewed not as a stand-alone cure but as a valuable adjunct to lifestyle and pharmacological interventions [152].

However, applying antioxidants in chronic syndromes like PCOS faces challenges. Clinical results have been inconsistent: while small trials and animal studies often report improvements, many larger human trials (in PCOS and other diseases) fail to show clear benefits on hard outcomes such as disease progression or pregnancy rates. For example, meta-analyses highlight that optimal dosing and long-term effects of antioxidants in PCOS remain undefined. Major obstacles include poor bioavailability and tissue-targeting of many compounds [153]; high doses are often needed to achieve cellular effects, raising safety concerns. The oxidative network itself is complex, with multiple ROS species and redox-sensitive pathways in delicate balance – supplementing exogenous antioxidants may unpredictably disrupt signaling, and some agents can even act as pro-oxidants at high concentrations [[154], [155], [156]]. Moreover, the field lacks universally accepted biomarkers of oxidative stress, complicating the assessment of therapeutic impact. In sum, while antioxidant supplementation shows mechanistic promise, translating it into clinical practice is hindered by these pharmacokinetic and pharmacodynamic limitations.

Established PCOS management focuses first on lifestyle and metabolic control. International guidelines designate weight management (diet, exercise, behavioral changes) as first-line therapy [157]. Dietary interventions such as low–glycemic-index (GI) diets have proven benefits: randomized trials show that low-GI regimens significantly lower fasting insulin and insulin resistance in PCOS, even without calorie restriction [158]. Such diets reduce insulin-driven ROS generation and help lower androgen levels indirectly. Likewise, regular physical activity improves mitochondrial efficiency and insulin sensitivity, resulting in less adiposity-driven ROS. Behavioral measures (stress reduction, sleep hygiene) also mitigate stress hormones and inflammation that can exacerbate oxidative stress. These lifestyle approaches are known to improve reproductive and metabolic outcomes in PCOS, and their antioxidant effect (e.g. via increased antioxidant enzyme expression) likely contributes to their efficacy.

Pharmacological treatments address hormonal and metabolic derangements: metformin remains a cornerstone for insulin resistance and hyperinsulinemia, both improving ovulation and indirectly reducing insulin-induced ROS production [159]. Oral contraceptives and anti-androgens chiefly treat hyperandrogenic symptoms but have no effect on insulin resistance or oxidative stress per se. Ovulation induction agents (clomiphene, letrozole, gonadotropins) are used for infertility but do not address systemic metabolic abnormalities [160]. In this context, antioxidants are best viewed as complementary rather than alternative therapies. By integrating antioxidants with diet/exercise and drugs, clinicians aim to both correct core endocrine/metabolic defects and blunt the oxidative burden. For example, adding NAC or lipoic acid to metformin and lifestyle therapy often yields better glycemic and ovulatory outcomes than conventional measures alone [161]. No standard antioxidant protocol exists, so current practice involves individualized supplementation alongside proven treatments.

Despite advances, important gaps remain in understanding and targeting ROS in PCOS. Upstream regulators of ROS overproduction in PCOS – such as specific genetic variants, signaling kinases or mitochondrial genome alterations – are not fully elucidated [162]. Application of cutting-edge tools (CRISPR/Cas9 editing, proteomics, single-cell RNA-Seq) could identify novel nodes in the redox network that trigger oxidative stress in PCOS ovaries or adipose tissue [2,163]. On the therapeutic front, large randomized controlled trials are urgently needed to validate antioxidant interventions. Multi-center trials examining clinical endpoints (pregnancy rates, metabolic syndrome) over longer durations would determine whether the improvements in redox markers translate into tangible health benefits. Novel delivery systems (e.g. nanoparticle carriers targeting the ovary or mitochondria) warrant exploration to improve antioxidant bioavailability and specificity. Finally, PCOS is highly heterogeneous: future research should move toward precision medicine approaches that integrate individual genetic and metabolic profiles. Developing panels of oxidative stress biomarkers could allow tailoring of antioxidant therapy to those patients most likely to benefit. In this personalized paradigm, treatments would be optimized not only for symptom control but for interrupting the patient's unique pathogenic ROS circuitry.

In conclusion, ROS lie at the heart of PCOS pathogenesis, where elevated ROS levels damage oocytes and GCs—impairing follicular development and maturation and contributing to poor oocyte quality, disrupted ovulation and infertility—while also undermining insulin signaling and driving androgen overproduction to integrate metabolic and endocrine dysfunctions. Consequently, addressing oxidative stress through a comprehensive strategy—combining lifestyle interventions (diet, exercise, weight management), insulin-sensitizing and antiandrogenic pharmacotherapy, and evidence-based antioxidant supplementation—offers the greatest potential to improve long-term outcomes. Rigorous future trials should delineate optimal antioxidant agents, dosages and timing to ensure precise, effective clinical application in PCOS management.

## 6 Conclusion

This study underscores the pivotal role of ROS in the pathogenesis of PCOS, a common endocrine and metabolic disorder affecting women of reproductive age. Elevated ROS levels disrupt ovarian function, contribute to insulin resistance, and exacerbate hyperandrogenism, thereby influencing both reproductive and metabolic aspects of PCOS. Obesity and mitochondrial dysfunction further amplify oxidative stress, complicating the clinical presentation of PCOS. Accurate detection of ROS through various methodologies facilitates early diagnosis, effective disease monitoring, and prognostic assessments. Therapeutic interventions targeting oxidative stress, including antioxidant supplementation and lifestyle modifications, have shown promise in mitigating PCOS symptoms and improving patient outcomes. However, the precise regulatory mechanisms governing ROS production remain incompletely understood, and robust clinical trials evaluating antioxidant therapies are limited. Future research should employ advanced technologies to elucidate these mechanisms, conduct large-scale studies to validate therapeutic strategies, and develop personalized treatment approaches to enhance the management of PCOS.

## CRediT authorship contribution statement

**Huanju Liu:** Writing – review & editing, Writing – original draft, Methodology, Investigation, Funding acquisition, Formal analysis, Data curation, Conceptualization. **Lihao Jin:** Writing – review & editing, Visualization, Validation, Software, Conceptualization. **Xiaoya Wang:** Software, Data curation. **Junling Shi:** Software, Resources. **Yujie He:** Data curation. **Ningxia Sun:** Writing – review & editing, Supervision, Resources, Funding acquisition. **Fu Yang:** Writing – review & editing, Writing – original draft, Validation, Supervision, Resources, Project administration, Conceptualization.

## Declarations Ethics approval and consent to participate

Not applicable.

## Funding

The article was supported by the 10.13039/501100001809National Natural Science Foundation of China (82271662), National key research and development program (2022YFA1303900), Shanghai Health Science Academic Leader Development Program (2022XD003), and Yizhang Talent Plan (JCYZRC-D-023).

## Declaration of Competing Interest

The authors declare that they have no known competing financial interests or personal relationships that could have appeared to influence the work reported in this paper.

## Data Availability

No data was used for the research described in the article.

## Abbreviations

ADMA-DDAH1: Asymmetric Dimethylarginine-Dimethylarginine Dimethylaminohydrolase 1
AKT: Protein Kinase B
ALAS2: Aminolevulinate Synthase 2
ALOX15: Arachidonate 15-Lipoxygenase
AMH: Anti-Müllerian Hormone
AMPK: AMP-activated Protein Kinase
BMI: Body Mass Index
BNIP3: BCL2 Interacting Protein 3
BPA: Bisphenol A
BSTJF: Bu-Shen-Tian-Jing Formula
CD44: Cluster of Differentiation 44
CHO cells: Chinese Hamster Ovary cells
CYP17A1: Cytochrome P450 17α-hydroxylase
DCFH-DA: 2′,7′-dichlorodihydrofluorescein diacetate
DHEA: Dehydroepiandrosterone
DHT: Dihydrotestosterone
ERK1/2: Extracellular signal-regulated kinase 1/2
ER-FOXO1-ROS–NF–κB axis: Endoplasmic Reticulum-Forkhead box protein O1-Reactive Oxygen Species-Nuclear Factor-kappa B axis
FF: Follicular Fluid
FSH: Follicle-stimulating Hormone
GDF15: Growth Differentiation Factor 15
GCs: Granulosa Cells
GLUT4: Glucose Transporter Type 4
GnRH: Gonadotropin-releasing Hormone
GPx: Glutathione Peroxidase
GSH: Glutathione
H_2_O_2_: Hydrogen Peroxide
Hcy: Homocysteine
HFD: High-Fat Diet
HIF-1α: Hypoxia-Inducible Factor 1-alpha
HO-1: Heme Oxygenase-1
HPO: Hypothalamic-Pituitary-Ovarian axis
IL-1β: Interleukin-1 beta
IL-6: Interleukin-6
JNK: c-Jun N-terminal Kinase
KGN cells: Human Ovarian Granulosa Tumor Cell Line
LDL-C: Low-Density Lipoprotein Cholesterol
LH: Luteinizing Hormone
LPS: Lipopolysaccharide
LRRC17: Leucine Rich Repeat Containing 17
MAPK: Mitogen-Activated Protein Kinase
MDA: Malondialdehyde
MSS51: Mitochondrial Ribosomal Protein S51
mtDNA: Mitochondrial DNA
mTOR: Mammalian Target of Rapamycin
NAD^+^: Nicotinamide Adenine Dinucleotide
NAC: N-Acetylcysteine
NADPH oxidase: Nicotinamide Adenine Dinucleotide Phosphate Oxidase
NF: Nuciferine
NF-κB: Nuclear Factor Kappa-light-chain-enhancer of activated B cells
NIH: National Institutes of Health
NLRP3: NOD-like Receptor Family Pyrin Domain Containing 3
NMN: Nicotinamide Mononucleotide
NO: Nitric Oxide
NOX2: NADPH Oxidase 2
NR: Nicotinamide Riboside
Nrf2: Nuclear factor erythroid 2-related factor 2
O_2_^−^: Superoxide Anion
·OH: Hydroxyl Radical
PCOS: Polycystic Ovary Syndrome
PDK4: Pyruvate Dehydrogenase Kinase 4
PHLPP1: PH Domain Leucine-rich repeat Protein Phosphatase 1
PI3K: Phosphoinositide 3-Kinase
PPAR-γ: Peroxisome Proliferator-Activated Receptor Gamma
PSTi8: Pancreastatin inhibitor 8
RAGE: Receptor for Advanced Glycation End-products
ROS: Reactive Oxygen Species
SHBG: Sex Hormone-Binding Globulin
SIRT1: Sirtuin 1
SIRT3: Sirtuin 3
SLC7A11: Solute Carrier Family 7 Member 11
SOD: Superoxide Dismutase
SOX2: SRY-Box Transcription Factor 2
SPRY4: Sprouty RTK Signaling Antagonist 4
SREBF2: Sterol Regulatory Element Binding Transcription Factor 2
SULT: Sulfotransferase
TAC: Total Antioxidant Capacity
TET: Ten-Eleven Translocation
TFRC: Transferrin Receptor
TIGAR: TP53-Induced Glycolysis and Apoptosis Regulator
TLR4: Toll-like Receptor 4
TNF-α: Tumor Necrosis Factor-alpha
TPEN: N,N,N′,N'-Tetrakis (2-pyridylmethyl) ethylenediamine
UPR^mt^: Mitochondrial Unfolded Protein Response
VEGFA: Vascular Endothelial Growth Factor A
YKL-40: Chitinase-3-like protein 1
YTHDF2: YTH N6-Methyladenosine RNA Binding Protein 2
